# Predictive value of geriatric nutritional risk index in patients with pancreatic cancer: a meta-analysis

**DOI:** 10.3389/fnut.2025.1464447

**Published:** 2025-07-29

**Authors:** Yaqi Hua, Yi Yuan, Chen Zhou, Liping Liu, Yan Hu, Ping Tu, Dongying Li

**Affiliations:** ^1^Department of Intensive Care Unit, The 2nd Affiliated Hospital, Jiangxi Medical College, Nanchang University, Nanchang, China; ^2^School of Nursing, Jiangxi Medical College, Nanchang University, Nanchang, China; ^3^School of Nursing, University of South China, Hengyang, Hunan, China; ^4^Nursing Department, The 2nd Affiliated Hospital, Jiangxi Medical College, Nanchang University, Nanchang, China; ^5^Wound Ostomy Clinic, The 2nd Affiliated Hospital, Jiangxi Medical College, Nanchang University, Nanchang, China; ^6^Department of Post Anesthesia Care Unit, The 2nd Affiliated Hospital, Jiangxi Medical College, Nanchang University, Nanchang, China

**Keywords:** geriatric nutritional risk index, pancreatic cancer, prognosis, survival, meta-analysis

## Abstract

**Objective:**

While growing evidence supports the Geriatric Nutritional Risk Index (GNRI) as a prognostic indicator for various cancers, its predictive value in pancreatic cancer remains unclear. This meta-analysis systematically evaluates GNRI’s ability to predict postoperative complications and long-term outcomes in pancreatic cancer patients.

**Methods:**

We conducted a comprehensive literature search across nine databases (Web of Science, PubMed, Embase, Cochrane Library, Scopus, WanFang, CNKI, VIP, and SinoMed) through June 1, 2025. Hazard ratios (HRs) with 95% confidence intervals (CIs) were used to assess overall survival (OS), while risk ratios (RRs) with 95% CIs evaluated postoperative complications.

**Results:**

From 233 initially identified studies, 10 met inclusion criteria (n = 2,003 patients). Pooled analysis revealed that lower GNRI significantly predicted worse OS (HR = 1.92, 95% CI 1.54–2.41, *p* < 0.0001) and higher postoperative pancreatic fistula (POPF) incidence (RR = 0.18, 95% CI 0.08–0.43, *p* < 0.001). No significant association was found between GNRI and post-pancreatectomy hemorrhage (PPH) (RR = 0.21, 95% CI 0.03–1.53, *p* = 0.13).

**Conclusion:**

GNRI shows promise as a clinically useful predictor of OS and POPF in pancreatic cancer patients. However, these findings require validation through prospective multicenter studies.

**Systematic review registration:**

Identifier CRD42023409362.

## Introduction

Pancreatic cancer (PC) is the third leading cause of cancer-related death in the US (1). In 2020, there have been about 466,000 deaths worldwide from pancreatic cancer. According to the American Cancer Society, 64,050 new cases and 50,550 new deaths of pancreatic cancer occurred in the United States in 2023 (2). Even though pancreatic cancer only represents 2.5% of all cancers, it is characterized by an insidious onset that typically renders it asymptomatic in its early stages, resulting in a late-stage diagnosis (3). As such, it is associated with a poor prognosis, with a 5-year survival rate of only 12% (2). Currently, numerous investigations have reported the prognostic indicators for pancreatic cancer, with emphasis on histochemical and molecular biological techniques such as CA19-9, circulating tumor DNA, and MicroRNA (4–6). Nonetheless, in clinical practice, these biomarkers are limited in application due to a lack of methodological standardization and quality control (7). Therefore, it is urgent to find convenient, high-speed, and inexpensive prognostic factors for pancreatic cancer. The Geriatric Nutritional Risk Index (GNRI) is a valuable and simple tool for screening malnutrition, which includes two items: body weight and serum albumin, comprehensively reflecting the body’s nutritional status (8, 9). The nutritional status of patients to some extent reflects the progression of the disease (10). GNRI has been used in various clinical settings and has shown good prognostic value in tumor patients (11). Furthermore, its prognostic role has been verified in several types of cancers, such as urological cancers, gastrointestinal malignancy, and Non-Small Cell Lung Cancer, by meta-analyses (12–14). Nevertheless, the predictive potential of GNRI in pancreatic cancer has yet to be systematically evaluated. Thus, the objective of this meta-analysis is to examine the impact of GNRI on the prognosis of pancreatic cancer.

## Materials and methods

This meta-analysis was conducted in accordance with the PRISMA guidelines and registered in the International Prospective Register of Systematic Reviews (PROSPERO) under registration number CRD42023409362.

### Search strategy

A systematic literature search was performed in the following databases up to June 1, 2025: Web of Science, PubMed, Embase, Cochrane Library, Scopus, WanFang, CNKI, VIP, SinoMed. Search terms included: “pancreas,” “cancer,” and “geriatric nutritional risk index.” The PubMed search strategy was as follows: (neoplasms OR carcinoma OR cancer OR tumor OR malignancy OR adenoma OR neoplasm OR cancers) AND (pancreatic OR pancreas) AND (geriatric nutritional risk index OR GNRI). To ensure comprehensive coverage, we also manually screened the references of retrieved articles for additional eligible studies.

### Eligibility criteria

Articles meeting the following criteria were included: (1) P (Participant): Patients with pathologically confirmed pancreatic cancer; (2) I (Intervention): Patients have had a high GNRI index, GNRI calculated using the formula: GNRI = [1.489 × serum albumin (g/L)] + [41.7 × (current weight/ideal weight (kg))]; (3) C (Comparison): Patients have had a low GNRI index; (4) O (Outcome): Overall survival (OS) (with hazard ratio [HR] and 95% confidence interval [CI]); Postoperative complications (with risk ratio [RR] and 95% CI); (5) S (Study design): Retrospective or prospective studies with full-text availability. We excluded reviews, conference abstracts, case reports, letters, comments, meta-analyses, studies lacking complete data for analysis.

### Data abstraction

Two independent researchers used EndNote for literature management and screening. Extracted the following data: First author, study type, publication year, region, median follow-up, sample size, clinical staging, treatment, GNRI cutoff-value, outcomes (HR/RR with 95% CI). Resolved discrepancies through discussion or third-party arbitration until consensus was reached.

### Methodological quality assessment

Study quality was assessed using the Newcastle-Ottawa Scale (NOS), which evaluates: Selection (4 points), Comparability (2 points), Exposure/Outcome (3 points). A total score ≥ 7 indicated high-quality studies, while <7 indicated lower quality.

### Statistical analysis

The meta-analysis used Stata (version 13.0) to extract research data and generate the forest map. In our meta-analysis, the HRs and RRs with corresponding 95% CIs were combined to explore the relationship between the preoperative GNRI and OS or postoperative complications in pancreatic cancer patients. The heterogeneity was detected by the Q test. A random-effects model was applied if *p* < 0.1 or I^2^ > 50%; otherwise, a fixed-effects model was used. After combined analysis, it was considered statistically significant when *p* < 0.05. Sensitivity analysis was conducted to identify potential sources of heterogeneity and assess the influence of individual studies on the overall results. By using Begg’s test and Egger’s test to determine potential publication bias, when *p* < 0.05, it is considered that there is publication bias. If publication bias was detected, the trim-and-fill method was employed for adjustment and re-evaluation.

## Results

### Literature search and study characteristics

**Figure 1:**
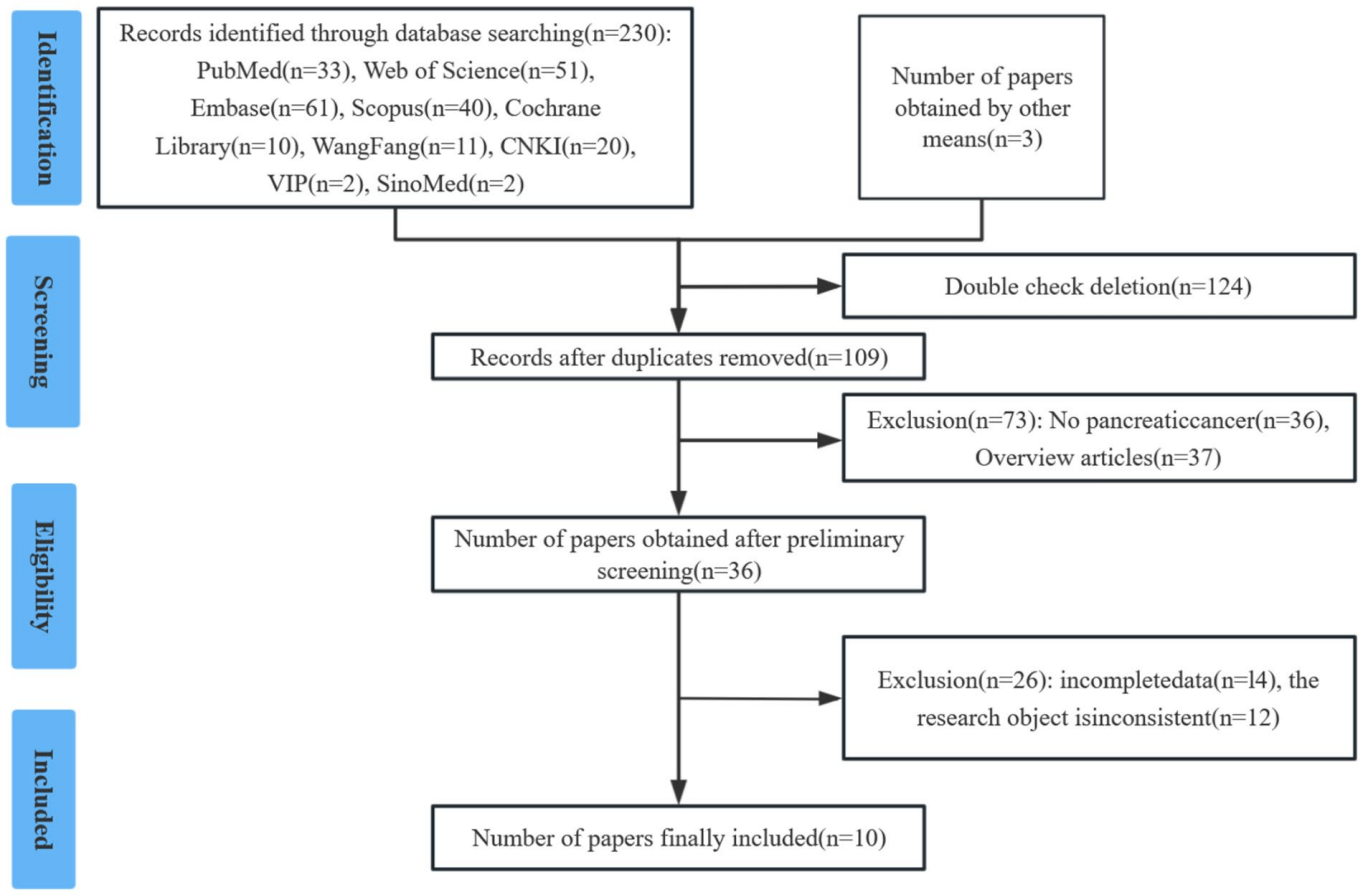
PRISMA flow diagram of study selection process.

The initial literature search yielded 233 potentially relevant studies. Following a systematic screening process, 10 studies involving a total of 2,003 cases were selected for final analysis (15–24) (Figure 1). The included studies comprised one prospective cohort study, nine retrospective cohort studies. Quality assessment using the predefined criteria revealed that all 10 studies scored ≥7 points, indicating high methodological quality and a low risk of bias. Table 1 presents the baseline characteristics and primary outcome measures of the included studies.

**Table 1:** Basic characteristics and quality evaluation of included studies.

Study	Design	Region	MFP (months)	Sample size	Age (years)	Clinical staging	Therapy	Cut-off value	Outcome	NOS (points)
Balzano G 2017 (15)	PS	Italy	64.8	296	68.9 (IQR: 14)	I–IV	Surgery	98	OS	7
Hu Siping 2019 (16)	RS	China	12.6	146	67.7 ± 5.8	I–IV	Surgery	100.2	OS	7
Funamizu N 2020 (1) (17)	RS	Japan	-	37	73 (35–82)	I–IV	Surgery	96	POPF	7
Funamizu N 2020 (2) (18)	RS	Japan	-	121	76.1 ± 2.0 (10) /70.8 ± 1.1 (111)	I–IV	Surgery	92	PPH	7
Hu SP 2020 (19)	RS	China	72.9	282	58.7 ± 13.5	I–IV	Surgery	98	OS	8
Itoh S 2021 (20)	RS	Japan	20.4	589	71 (63–77)	I–III	Surgery	98	OS	7
Sakamoto T 2021 (21)	RS	Japan	26.6	105	73.4 (65–84)	I–IV	Mixed	98	OS	8
Funamizu N 2022 (22)	RS	Japan	≤60	139	70 (34–89)	I–IV	Surgery	99	OS	7
Grinstead C 2022 (23)	RS	America	2.9	98	66 ± 9.8	III–IV	Mixed	98	OS	8
Zhang Bolin 2022 (24)	RS	China	-	190	≥65	I–IV	Surgery	98	PPH; POPF	7

PS, Prospective cohort study; RS, Retrospective cohort study; POPF, Postoperative pancreatic fistula; PPH, Post-pancreatectomy hemorrhage; OS, Overall Survival; NOS, The Newcastle-Ottawa Scale.

### GNRI and overall survival

**Figure 2:**
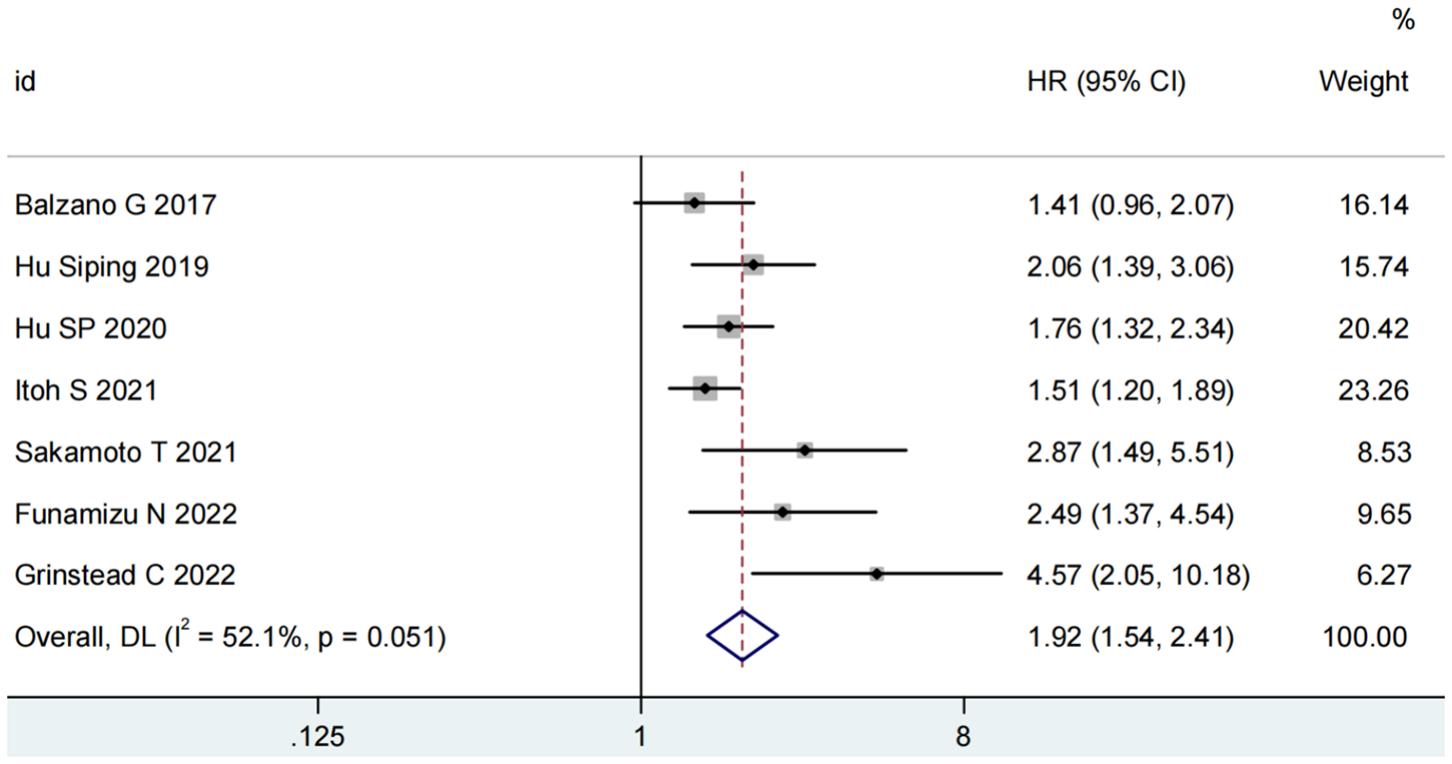
Forest plots for the meta-analysis regarding the association between GNRI and OS in patients with pancreatic cancer.

Seven studies involving 1,655 patients examined the relationship between GNRI and overall survival (OS) (15, 16, 19–23). The pooled analysis demonstrated a significant association between low GNRI and poorer OS in pancreatic cancer patients (HR = 1.92, 95% CI 1.54–2.41, *p* < 0.0001), with substantial heterogeneity observed (I^2^ = 81%, *P* for Q-test = 0.05; Figure 2). To address potential confounding factors, we performed subgroup analyses stratified by cut-off value, sample size, primary therapy and publishing time. These subgroup analyses consistently identified GNRI as an independent prognostic factor for OS across all strata (Table 2).

**Table 2:** Stratification analysis of the meta-analysis for overall survival in patients with pancreatic cancer.

Factors	No. of studies	No. of patients	Effects model	HR (95%CI)	*p*	Heterogeneity	
						I^2^ (%)	*P*_Q_
Overall	7 (15, 16, 19–23)	1,655	REM	1.92 (1.54–2.41)	<0.0001	81	0.05
Cut-off value							
98	5 (15, 19–21, 23)	1,370	REM	1.68 (1.44–1.97)	<0.0001	61	0.04
99	1 (22)	139	-	2.49 (1.37–4.54)	0.003	-	-
100.2	1 (16)	146	-	2.87 (1.49–5.51)	0.002	-	-
Sample size							
≤200	4 (16, 21–23)	488	FEM	2.50 (1.90–3.30)	<0.00001	10	0.34
≥200	3 (15, 19, 20)	1,167	FEM	1.57 (1.33–1.84)	<0.0001	0	0.60
Primary therapy							
Surgery	5 (15, 16, 19, 20, 22)	1,452	FEM	1.67 (1.44–1.93)	<0.0001	10	0.35
Mixed	2 (21, 23)	203	FEM	3.47 (2.09–5.76)	<0.00001	0	0.37
Publishing time							
≤2020	3 (15, 16, 19)	724	FEM	1.73 (1.42–2.11)	<0.0001	0	0.40
>2020	4 (20–23)	931	REM	2.43 (1.47–4.02)	0.001	72	0.01

FEM, fixed-effects model; REM, random-effects model.

### GNRI and postoperative pancreatic fistula

**Figure 3:**
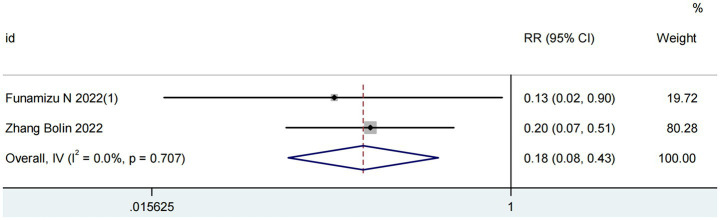
Forest plots for the meta-analysis regarding the association between GNRI and POPF in patients with pancreatic cancer.

Two studies involving 227 patients evaluated the predictive value of GNRI for postoperative pancreatic fistula (POPF) (17, 24). As illustrated in Figure 3, the analysis revealed no significant heterogeneity (I^2^ = 0%; *P* for Q-test = 0.71). The pooled results demonstrated that higher GNRI levels were significantly associated with reduced POPF incidence in pancreatic cancer patients (RR = 0.18, 95% CI 0.08–0.43, *p* < 0.001).

### GNRI and post-pancreatectomy hemorrhage

**Figure 4:**
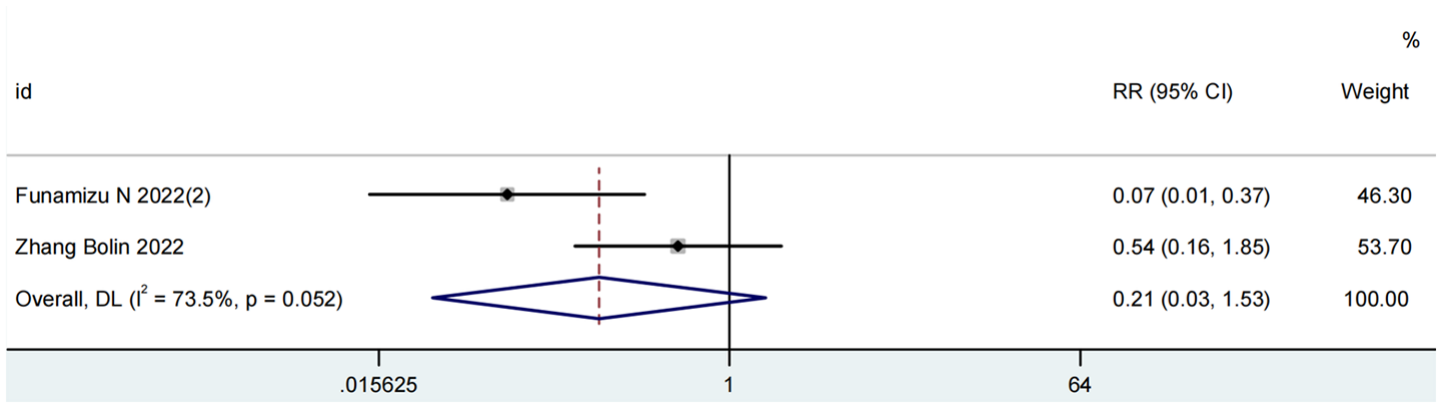
Forest plots for the meta-analysis regarding the association between GNRI and PPH in patients with pancreatic cancer.

Two studies comprising 311 patients assessed the prognostic value of the GNRI for post-pancreatectomy hemorrhage (PPH) (18, 24). As depicted in Figure 4, significant heterogeneity was observed (I^2^ = 73%; *P* for Q-test = 0.05). The meta-analysis revealed no significant association between elevated GNRI levels and reduced PPH incidence in pancreatic cancer patients (RR = 0.21, 95% CI 0.03–1.53, *p* = 0.13).

### Sensitivity analysis

**Figure 5:**
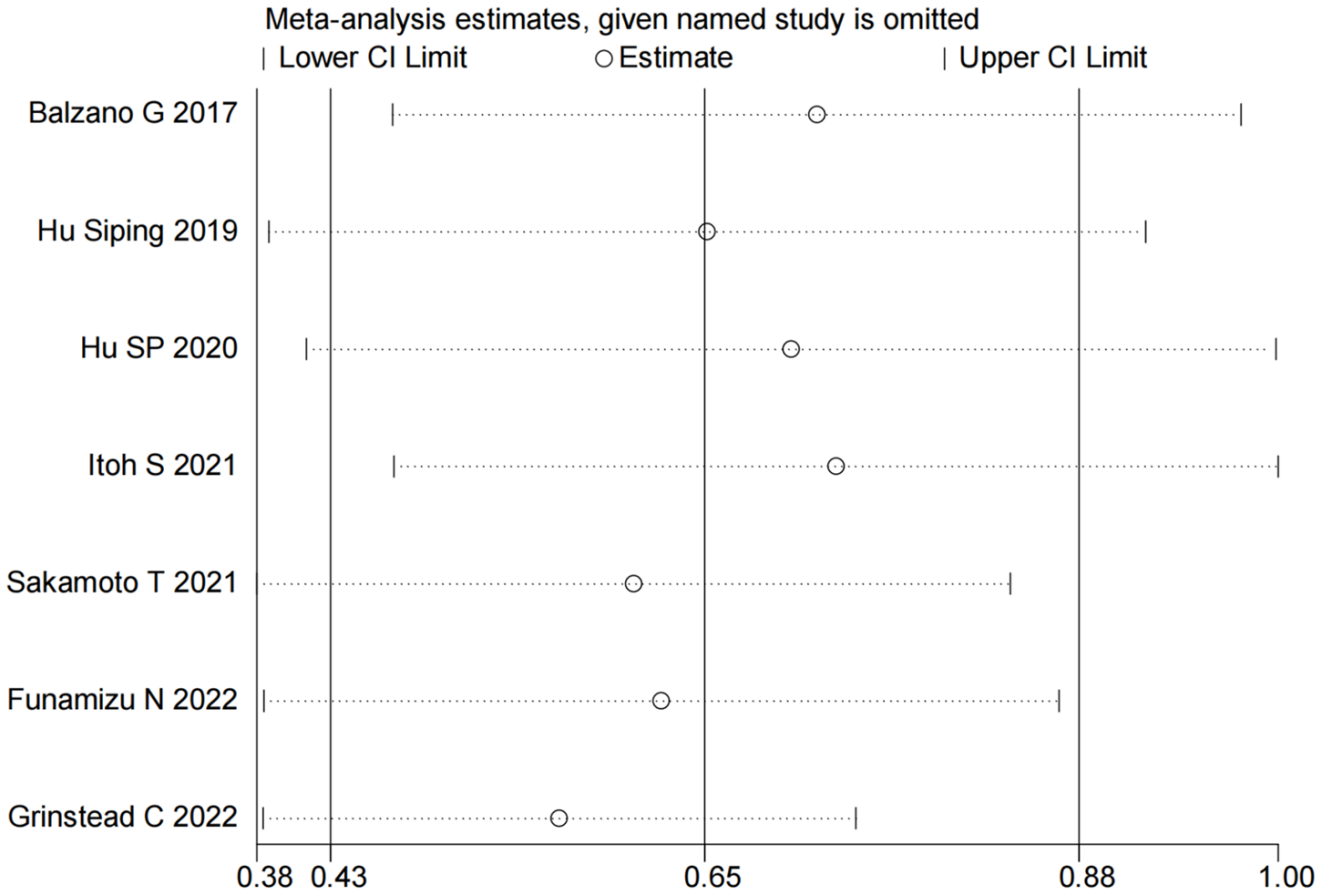
Sensitivity analysis for the association between GNRI and OS.

We performed sensitivity analyses to assess the robustness of the association between GNRI and OS. Specifically, each included study was sequentially removed from the meta-analysis to evaluate its individual impact on the pooled results. The sensitivity analysis demonstrated that the exclusion of any single study did not significantly alter the overall effect estimate (Figure 5), indicating stable and reliable findings regarding the prognostic value of GNRI for OS in pancreatic cancer patients.

### Publication bias

**Figure 6:**
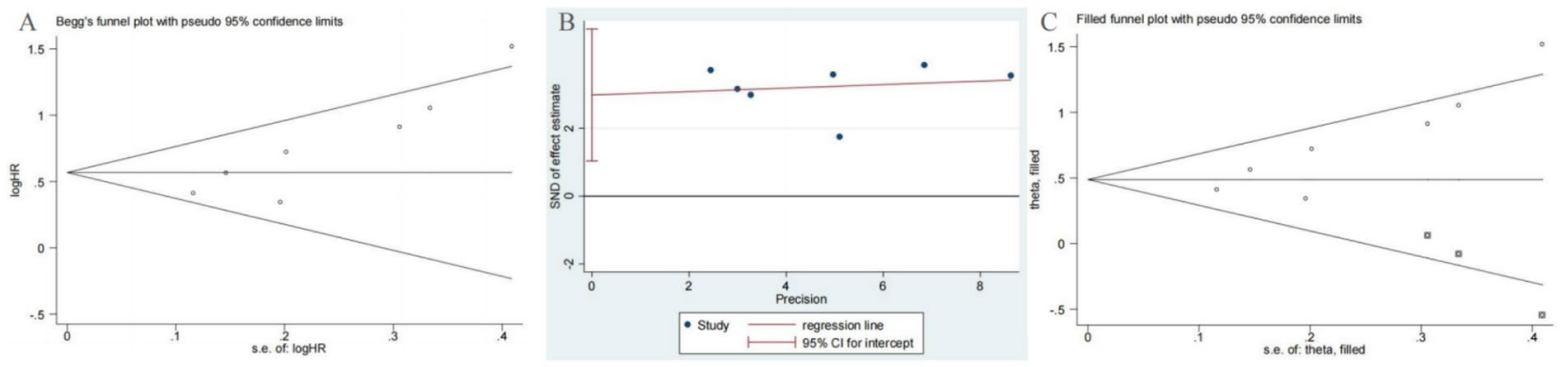
Plots for publication bias test in meta-analysis for overall survival. **(A)** Begg’s funnel plot; **(B)** Egger’s publication bias plot; **(C)** The trim-and-fill methods.

In our meta-analysis of OS, both Begg’s test (*p* = 0.007) and Egger’s test (*p* = 0.011) indicated the presence of significant publication bias (Figures 6A,B). To address this potential bias, we applied the trim-and-fill method. This adjustment resulted in the imputation of three additional studies to achieve symmetry in the funnel plot (Figure 6C). Importantly, the corrected hazard ratio remained statistically significant (HR = 1.65, 95% CI 1.29–2.11, *p* < 0.001), confirming the robustness of our primary findings.

## Discussion

The advancement of tumor nutrition theory has established a significant association between nutritional status and cancer prognosis, leading to the clinical application of various nutritional risk assessment tools. Currently, the primary nutritional indices include: Prognostic Nutritional Index (PNI), Nutritional Risk Index (NRI), Geriatric Nutritional Risk Index (GNRI), Controlling Nutritional Status (CONUT) score (25–27). Among these, GNRI has demonstrated superior predictive performance. Wang et al. compared these four indices in 192 esophageal cancer patients and found GNRI to be the most effective prognostic indicator for perioperative management (28). Originally developed by Bouillanne et al. to assess morbidity and mortality in hospitalized elderly patients (9), GNRI has since been adapted for prognostic evaluation in chronic diseases (11, 26). GNRI’s clinical utility stems from its composite nature, incorporating both serum albumin levels and body weight measurements to provide a dynamic, objective assessment of nutritional status (29, 30). Serum albumin serves as a crucial prognostic biomarker in oncology, with demonstrated value in predicting patient survival across multiple cancer types. Its clinical importance is evidenced by its incorporation into standard cancer staging systems (31, 32). As a multifunctional indicator, serum albumin reflects both nutritional status and systemic inflammation (33). Hypoalbuminemia (low serum albumin) signifies two clinically important pathological states: First, it indicates malnutrition, which compromises immune function and prolongs disease course (34, 35); second, it reflects systemic inflammation, as inflammatory processes suppress hepatic albumin synthesis while increasing vascular permeability, thereby exacerbating albumin loss (35, 36). Notably, the inflammatory cytokines associated with hypoalbuminemia may directly promote tumor progression and correlate with poorer clinical outcomes (37). This relationship was quantitatively demonstrated by Yang et al. in a large-scale study (*n* = 82,061), which established a significant inverse linear correlation between serum albumin levels and cancer risk (38). Concurrently, weight loss—a key component of cancer cachexia diagnosis (39, 40)—contributes to metabolic dysregulation affecting carbohydrate, lipid, and protein metabolism (41, 42). These metabolic disturbances impair immune competence and tissue repair capacity, creating a vicious cycle that accelerates functional decline and worsens cancer prognosis (41–43). The combination of these albumin-related and weight-related pathophysiological mechanisms explains the consistent clinical observation that lower GNRI scores (incorporating both parameters) predict poorer outcomes. Building on these principles, Balzano et al. (15) achieved a milestone in pancreatic cancer research by successfully incorporating GNRI into a predictive scoring system for postoperative mortality.

Our meta-analysis incorporated 10 relevant studies comprising 2,003 pancreatic cancer patients. The results demonstrate that the GNRI serves as an independent prognostic factor for pancreatic cancer outcomes. Subgroup analyses stratified by cut-off value, sample size, primary therapy, and publication time consistently confirmed GNRI’s independent predictive value for OS across all subgroups. Furthermore, while GNRI was identified as an independent risk factor for POPF, it showed no statistically significant association with PPH. Sensitivity analyses confirmed the stability and reliability of these findings. Although we detected publication bias, trim-and-fill adjustment maintained the significant association between low GNRI and poor OS, supporting the robustness of our conclusions. These results suggest that GNRI may be a potential indicator for predicting postoperative complications and prognosis in patients with pancreatic cancer.

Several limitations should be acknowledged. First, the number of included studies was relatively small (*n* = 10), particularly for POPF and PPH analyses (*n* = 2), and further research is needed to explore the role of GNRI in these complications. Second, heterogeneity may exist due to variations in GNRI cutoff values, treatment protocols, and demographic characteristics across the included studies. Additionally, all studies were retrospective in design; therefore, future prospective randomized controlled trials are required to validate the predictive value of GNRI. Finally, Begg’s and Egger’s tests indicated potential publication bias, which may reflect the limited number of available studies and should be considered in future investigations.

## Conclusion

This study provides a comprehensive evaluation of the prognostic significance of the GNRI in pancreatic cancer. Our findings demonstrate a statistically significant association between reduced GNRI levels and adverse clinical outcomes, particularly in OS and POPF incidence. The current analysis offers robust clinical evidence supporting the utility of GNRI as a practical prognostic indicator for pancreatic cancer patients. However, these conclusions require validation through large-scale, multicenter prospective cohort studies to strengthen their clinical applicability.

## Data Availability

The original contributions presented in the study are included in the article/supplementary material, further inquiries can be directed to the corresponding author.
